# The new informatics of pandemic response: humanitarian technology, efficiency, and the subtle retreat of national agency

**DOI:** 10.1186/s41018-018-0036-5

**Published:** 2018-05-30

**Authors:** Christopher Wilson, Maria Gabrielsen Jumbert

**Affiliations:** 1 Department of Media and Communication, University of Oslo, Oslo, Norway; 2 The Peace Research Institute Oslo (PRIO), Oslo, Norway

**Keywords:** Humanitarian, Pandemics, ICTs, New media, Informatics, Global networks, Risk communication

## Abstract

Digital communication technologies play an increasingly prominent role in humanitarian operations and in response to international pandemics specifically. A burgeoning body of scholarship on the topic displays high expectations for such tools to increase the efficiency of pandemic response. This article reviews empirical uses of communications technology in humanitarian and pandemic response, and the 2014 Ebola response in particular, in order to propose a three-part conceptual model for the new informatics of pandemic response. This model distinguishes between the use of digital communication tools for diagnostic, risk communication, and coordination activities and highlights how the influx of novel actors and tendencies towards digital and operational convergence risks focusing humanitarian action and decision-making outside national authorities’ spheres of influence in pandemic response. This risk exacerbates a fundamental tension between the humanitarian promise of new technologies and the fundamental norm that international humanitarian response should complement and give primacy to the role of national authorities when possible. The article closes with recommendations for ensuring the inclusion of roles and agency for national authorities in technology-supported communication processes for pandemic response.

## Introduction

Recent decades have seen a dramatic rise in global pandemics. From the SARS pandemic in 2003, to Avian Influenza in 2006, H1N1 in 2009, Ebola in 2014, and the appearance of the Zika virus in Latin America in 2015, these developments are inextricably bound up in modern socio-technical developments and processes of globalization. Advances in global air travel, agricultural technology, urbanization, and pollution all facilitate the appearance and spread of contagious diseases (see Wolfe 2011; Ramalingam 2015). Simultaneously, new media and technologies have also come to play a profound role in the way that global pandemics are identified, traced, understood, managed, treated, and perceived. Digital communication technologies play an increasingly significant role in different aspects of global pandemic response, presenting novel opportunities to mitigate risks and enhance response efficiency. In doing so, they also confound traditional domains of information and communication practices in pandemic response (Mager 2009) and introduce a novel collection of international and transnational actors to areas that have traditionally been the purview of national authorities.

The capacity of digital communications tools to process, systematize, and make sense of large amounts of data has attracted the attention of practitioners, policy makers, and scholars alike (Brownstein et al. 2009; Tusiime and Byrne 2011; Wesolowski et al. 2014; Zwitter and Hadfield 2014; Meier 2011; Holeman et al. 2016), and has raised significant expectations regarding their use in pandemic response in particular (Odugleh-Kolev 2014). These expectations are countered by an emerging critical scholarship concerned with the novel risks that accompany humanitarian technologies (Sandvik et al. 2014, 2017; Comes 2016), how communication technologies impact power relationships between national and international actors in humanitarian crises (Burns 2014; Letouzé et al. 2015; McDonald 2016), and the ways in which new technologies are both constituted by, and contribute to reshaping social practices in a given society (Amicelle et al. 2015).

With the exception of work by Ihlen and Levenshus’ (2017) on technology in crisis and risk management and Roberts and Elbe’s (2017: 14) on syndromic surveillance systems, however, the role of digital communication tools in pandemic response has received scant critical attention. This article contributes to filling that gap by conceptualizing the broad variety of ways in which digital communication technologies are brought to bear in global pandemic response. A three-part conceptual framework for informatics is constructed on the basis of empirical examples from recent pandemic responses and contemporary policy debates, drawing on the 2014 Ebola response in particular. This model highlights how the application of new communication technologies in pandemic response is often accompanied by an influx of novel actors and convergence of previously distinct activities within single technological platforms or institutional operations. Applying a critical reading of knowledge politics to these dynamics emphasizes the potential of new technologies to complicate global pandemic response, and the associated risk of relocating decision-making and agency outside of national authorities’ spheres of influence.

This article is organized in five sections. Following this introduction, a second section describes the literature upon which the conceptual framework for pandemic informatics is developed. The third section presents that framework and describes each of its three components in detail. The fourth section notes two underlying dynamics that are consistently cited in conjunction with the use of digital communications in pandemic response: the introduction of novel actors and convergence of activities in single technological platforms and across organizational entities. The final section concludes by noting the significant challenges that this poses for effective coordination of pandemic response between national and international actors, and elaborates on the tendency of technologically driven informatics to decrease national authorities’ sphere of influence in pandemic response. Reflecting on seminal and watershed humanitarian policy for humanitarian coordination, the article concludes by suggesting that the roles and authority of national actors be explicitly designed in workflows for internationally coordinated pandemic response, and identifies preliminary measures through which this might be pursued.

## Sources and analytical framework

This conceptual exercise draws on descriptions of digital technology deployment in several contexts and from a variety of sources. In addition to academic research, this includes liberal reference to the so called “grey literature” produced by humanitarian implementing agencies, think tanks and volunteer groups, as well as descriptions of humanitarian communication in popular press and in online media maintained by civil society organizations and other commentators. While such sources are not subject to significant quality or review standards, and often lack the detail found in other types of literature, they tend to describe a much wider variety of activities, and do so without the significant time lag that accompanies peer reviewed research. Often, activities and initiatives described in organizational reports, popular media, or NGO blogposts represent novel combinations of activities only anticipated in scholarly work and add a depth and richness to the scope of activity conceptualized in this article.

The current analysis is grounded in response to the 2014 Ebola pandemic, as arguably the most globally active and thoroughly documented example of pandemic response. Accordingly, second section uses examples of digital technology innovation from the Ebola response to construct a preliminary framework, with an emphasis on the incentives and objectives for using technological tools and strategies. The third section will then present each component of that preliminary framework in detail, using examples from other pandemics and from response policy more generally to validate and refine the framework.

## Reconsidering communication and technology in the 2014 Ebola response

A great deal has been written about how communication technologies were used in the global 2014 response to West African Ebola, including academic articles (Odugleh-Kolev 2014; Tulenko 2014; Sacks et al. 2015; Sandvik et al. 2017; Harman and Wenham 2018), assessments of multinational and non-governmental response (ACAPS 2015; Adams et al. 2015; DuBois et al. 2015; Smith 2015), and case studies documenting specific instances of technology use or country communication processes (NetHope Global Broadband and Innovations Alliance 2014; ACAPS 2015; Levine et al. 2015). The most comprehensive overview is provided by Fast and Waugaman’s (2016) 140-page report for USAID, “Fighting Ebola with Information,” which provides a starting point for this article’s conceptual framework. After briefly describing the types of activities covered in Fast and Waugaman’s report, an argument is made for re-categorizing and assessing those activities according to the objectives they pursue, which provides an initial basis for the framework developed in this article.

Fast and Waugaman provide a thorough account of how information and technology were mobilized in the West African Ebola response, based on case analysis, literature review, and over 130 interviews. Their analysis includes a typology of technology tools commonly utilized in the response (94–95) and nine in-depth case studies of specific information flows that relied on digital tools to facilitate response (63–91). These case studies include descriptions of activities, technological tools, actors involved, outcomes, challenges, and objectives and provide a useful starting point for drawing conclusions about the breadth of activities utilizing digital communications tools.

Fast and Waugaman organize and present their case studies by virtue of the

differences the integration of digital technologies enabled, such as increasing the diversity of information flows (e.g. “up” for data collection, as well as horizontally among peer groups, and back “down” through feedback loops) among a greater plurality of actors (e.g., frontline health workers, citizens, governments, and “remote” responders) (10).

The organizing principle here is a spatial understanding of coordination across pandemic response. Connecting and coordinating actors that would otherwise not have exchanged information is certainly one of technology’s most prominent contributions. A close read of these case studies, however, reveals a number of additional functions and objectives and allows for a more nuanced assessment of their impact.

The Liberian SMS-based initiative, mHero, for example, was structured not only to connect frontline workers and governments, but also explicitly “aimed to strengthen the government’s health information system […] and to provide critical information to support health workers on the frontlines of the crisis” (63). The Ebola Community Action Platform (eCAP) did not aim only to connect response organizations and extension workers, but more accurate information on the state of contagion and response was quickly leveraged towards public health messaging via billboards, radio, posters, handouts, and “person-to-person drama activities at the community level” (70). These examples are notable not only for the breadth of actors they engage, but for the deliberate piggybacking of novel objectives and efficiencies, and the same dynamic is discernable across all of Fast and Waugaman’s nine case studies.

This matters. The way in which objectives are articulated and claimed has consequences for how responsibilities are allocated and priorities made in humanitarian response (Burns 2015; McDonald 2016). In much the same way as the power asymmetries implied by humanitarian technology are often eclipsed by a presumption of technology’s democratizing potential to re-distribute power (Sandvik et al. 2014: 11), Fast and Waugaman’s preliminary mapping of digital information flows posits connectivity as an immediate and obvious benefit to pandemic response, without assessing the ways in which it asserts and reinforces power relationships.

As the sociologists would have it, however, technological tools and political instruments “are less inert intermediaries than partly autonomous actants that contribute to orientating actors’ behaviours” (Amicelle et al. 2015). Novel connectivity in pandemic response inevitably structures relationships of power and responsibility in a messy field, asserting and assigning influence and roles in humanitarian response. Critical scholars have described these dynamics as “knowledge politics” (Burns 2014). The degree to which actors are able to influence these knowledge political assertions and assignments, we term informatics discretion, relies significantly on their participation, capacity and expertise in the types of information and communication modalities at issue (Elwood 2010). Reconceptualizing digital information flows in pandemic response according to their objectives allows for a close reading of the ways in which this occurs and the implications it has for pandemic response coordination.

A careful review of Fast and Waugaman’s nine case studies suggests at least three broad categories of objectives that manifest themselves consistently across different modes of connectivity. Digital communications in Fast and Waugaman’s case studies are leveraged to determine the way in which Ebola was spreading and the nature of risks posed by the pandemic, in order to coordinate activity among different types of response actors, including national authorities, international humanitarian aid workers and front-line health care providers, and in order to communicate with the general public regarding health risks and appropriate behavior to mitigate those risks.

In order to test and refine these broad categories, they were assessed in the context of the broader literature on West African Ebola response, and in the broader context of pandemic response and humanitarian policy, including grey literature and popular media, as described above. Convenience sampling and citation tracing were used to identify relevant academic literature, while scans of the websites of civil society groups identified in academic research were used to identify sources in grey literature and popular press. When distinct objectives or efficiencies were described in the context of digital information flows, these were grouped according to the above categories. Those categories were then refined into a three-part conceptual model of informatics in pandemic response, which is described in the following section.

## The new informatics of pandemic response

The previous section’s review of objectives and efficiencies driving the use of digital communication technologies in Ebola response suggested three broad categories: (a) *diagnostic efforts*, through which the characteristics and spread of infectious diseases are assessed in order to inform treatment and response; (b) *risk communication practices*, through which communities and individuals are informed about pandemic risks in an effort to mitigate those risks and curb contagion; and (c) *coordination processes*, through which different actors involved in pandemic response are allocated roles and responsibilities, in an attempt to maximize the efficiency of their work and avoid superfluous or parallel efforts. This section provides detailed description of these categories, validated and refined through reference to other contexts.

It should be noted that some of these categories recall established fields of study. Risk communication and coordination activities are relatively well delineated in scholarly work and policy documents (see Plough and Krimsky 1987; Akl et al. 2015, respectively), while our understanding of diagnostic efforts combines a variety of activities occurring across the spectrum of response, from health surveillance, to the identification of the pandemic and clarification of symptoms, to contact tracing and contagion modeling. It should be emphasized that our objective here is not to recount or re-conceptualize established fields of practice, but rather to sketch the different mechanisms and patterns through which technology and information are deliberately employed, and to question the consequences this has for governance and power relationships in pandemic response more generally.

### Diagnostics

This first component groups diagnostic activities related to collecting, systematizing, and processing information about a disease outbreak, and mapping its spread and associated needs, which we term *diagnostics*. This constitutes diagnosis at a societal level, and should not be confused with the medical diagnosis of individuals.

The analysis of information has been central to understanding, anticipating and responding to infectious diseases at least since John Snow became the “father of modern epidemiology” by drawing dots around a map of London water pumps during the Cholera epidemic in 1854 (Hempel 2006). In the humanitarian context, collecting information is central to the implementation of an efficient response, including situational information, needs assessment, and operational information (King 2005; Van de Walle et al. 2008). This corresponds with humanitarian practice which has traditionally assumed the “information imperative” to be central to the humanitarian imperative—that is, the need to collect as much relevant information as possible, to enhance evidence for decision-making basis and improve efforts to assist people suffering in crisis contexts (Bui et al. 2000; Darcy and Hofmann 2003; Miller et al. 2005; Saab et al. 2008).

Scholars have explored a number of ways in which technology improves humanitarian diagnostics, including the use of mobile phone network data for human mobility mapping and contact tracing (Tatem et al. 2009; Aslam et al. 2014; Wesolowski et al. 2014; Gittelman et al. 2015; Bharti et al. 2015; Bengtsson et al. 2015), and big data or social media scraping for contagion modeling (Brownstein et al. 2009; Chunara et al. 2012). Such techniques allow humanitarian actors to extract information, diagnosing needs and epidemiological trends without direct contact with affected populations.

Other applications of digital media for diagnostic activities introduce completely different actors. Novel initiatives in response to the Ebola pandemic in 2014–2016 include hackathons organized in western capitals to map resources for West African response, and western non-governmental organizations (NGOs) that provide communication-based outbreak models to multinational humanitarian organizations (Sangokoya 2014; Moore 2015; Dittus et al. 2016) or build geographic information system (GIS) maps for national authorities (Timo Lüge 2014). Early work is even underway to develop artificial intelligence responses to combat the spread of infectious diseases, by using multiple sources of publically available data to algorithmically predict the appearance and spread of disease (Barron 2014).

Such innovative approaches are compelling and have attracted an understandable amount of interest and optimism, not in the least due to perceived gains in efficiency. Novel collaborations and web-based health surveillance systems have regularly been quicker than the World Health Organization (WHO) to publish reports on epidemic outbreaks (Anema et al. 2014: 1036-1037), and Jennifer Gardy, a senior scientist at the British Columbia Centre for Disease Control, has argued that epidemics are always essentially a race between the spread of info and spread of virus, and that technology has given scientists a critical edge (Edmunds 2013).

Whether or not such approaches are inherently more efficient, they are noteworthy for their inclusion of novel actors. This is perhaps most apparent in web-based initiatives such as wiki systems, which are used to facilitate collaborative pandemic modeling among geographically disparate scientists (Kno.e.sis 2014), or the Humanitarian Data Exchange, which through the leadership of a traditional humanitarian agency, was used to coordinate data sharing between a diverse group of actors, including local NGOs and remote volunteer mapping communities (Verhulst 2014; Fast and Waugaman 2016: 90). Though not solely dependent on digital media, a similar dynamic is visible in the proliferation of global health networks, such as GOARN (Global Outbreak Alert and Response Network) and ProMED (Program for Monitoring Emerging Diseases), which combine the efforts of national and international civil society and regulatory bodies, to surveil health risks internationally through a mix of traditional and digital media, and which to some degree eclipse the traditional roles of international and national health regulatory bodies (Ramalingam 2015: 9–13).

### Risk communication

The second component we identify is *risk communication*, which we understand as the processes of communicating the risks associated with a pandemic outbreak, primarily to relevant and potentially affected publics. It is a widely recognized field of practice defined by the American National Academy of Sciences as:

an interactive process of exchange of information and opinion among individuals, groups, and institutions. It involves multiple messages about the nature of risk and other messages, not strictly about risk, that express concerns, opinions, or reactions to risk messages or to legal and institutional arrangements for risk management. (cited in Covello et al. 2001: 382–383).

The interactive character referenced in that definition is the subject of some debate. In their Handbook on Global Health Communication, editors Waisbord and Obregon argue that the field of global health communication is characterized by a theoretical split, which places a preference for behavior change and unidirectional approaches against critical theories of participatory engagement (Waisbord and Obregon 2012: 7–33). Civil society and practitioner rhetoric tends to link participatory communicative models with technological advances, framed in normative terms that are highly critical of traditional, less participatory approaches in the humanitarian and development sectors (Chao 2014). This normative perspective is also assumed by several scholars (Kaiser 2000; Abraham 2011; Maxwell et al. 2011; Gillman 2014; Özdamar and Ertem 2015; Madianou et al. 2016), and participatory approaches to risk communication have been argued to improve outcomes and efficiency. Odugleh-Kolev (2014) argues that a structural and interactive understanding of risk communication is particularly important in pandemic response, where coordinated, functional, and systemic communication is implied in activities such as assessing transmission risk and mapping contagion patterns in rural communities (243). Participatory approaches to risk communication tend to incorporate diagnostic activities, since digital media technologies facilitate simultaneous broadcast and collection of humanitarian information, at marginal cost, as will be discussed below.

For clarity, this analysis focuses on risk communication implemented nationally or sub-nationally, targeting communities potentially affected by pandemics, and disregard the international role of for instance social media in risk communication. In doing so, we can identify at least three ways in which digital media are used intentionally to bolster risk communication strategies. Again, responses to the Ebola outbreak of 2014 are illustrative:

Firstly, digital media was expected to *expand the reach and interactivity* of risk communications. The use of new media technologies, particularly mobile phones, is often expected to dramatically increase the reach of risk communication to rural and remote communities, as well as front line health providers. Mobile penetration rates in developing countries were estimated to have surpassed 90% at the time of the Ebola outbreak (Wesolowski et al. 2014), and a review of Ebola response in Nigeria cited mobile-delivered training to health workers as “vital” for promptly declaring the country “free of Ebola” (West 2015: 10). Digital media also allow for scaled co-creation of health communication content, such as programs to produce videos together with youth infected with Ebola in Sierra Leone, as a means of addressing stigmatization and developing trust networks among affected populations (Zuckerman 2014).

Secondly, digital media allow for *feedback and interaction* to be integrated into risk communications. A project called U-Report, led by the United Nations Children’s Fund (UNICEF), combines mobile phone-based surveys with opportunities for user feedback and was used to channel infection reports and concerns regarding public health activities in Liberia during the Ebola outbreak (Muah et al. 2014). Similarly, the international conglomerate IBM combined outgoing radio communications with interactive short messaging system (SMS) functionality to implement interactive risk communications surrounding Ebola in Sierra Leone (Bell, 2014). Some studies suggest that these affordances were particularly effective when targeting health communication to specific sub-groups (Ems and Gonzales 2015).

Finally, digital media are sometimes expected to *obviate institutional and resource limitations* on the development of media platforms and content production for public health authorities. That digital media is faster and cheaper to produce and disseminate than paper is widely recognized. In addition, participatory content creation such as the Sierra Leone video program described above can effectively outsource some degree of communications work, as do content creation efforts which move beyond specific communities and aim to engage “the crowd” in generating content. Such approaches have been pursued in generating both platforms and content for risk communication, either through international volunteer communities or institutionalized global health networks, or initiated by third parties, as in a series of hackathons organized in New York to develop mobile apps for spreading information about Ebola in affected countries (Sangokoya 2014).

### Coordination

The third component we identify is coordination, which is a persistent challenge and polemic in the humanitarian sector (Stephenson 2005; Bisri 2016). The introduction of new actors and new technologies promise to mitigate this challenge, even as they contribute to it in novel ways. Ramalingam (2015) notes that the contemporary governance system for international diseases incorporates at least five different types of actors (Intergovernmental organizations, National governmental organizations, Non-governmental organizations, Private foundations, and Public/private partnerships and consortia), whose activities are marked by competition and lack of collaboration (ibid 5–6). He argues that this institutional disarray, when coupled with other socio-economic developments and trends of globalization, poses a threefold governance challenge to global health: the challenge of capitalizing on the diversity of actors, the challenge of bringing traditional actors up to speed with innovative organizational and technical approaches, and balancing the incentives and responsibilities of individual countries in treating diseases that do not respect borders (ibid 6).

Simultaneously, timely, accurate, and appropriate information is widely regarded as a cornerstone for effective humanitarian coordination (Bui et al. 2000; Miller et al. 2005; Saab et al. 2008) and digital media are often expected to dramatically enhance the coordination potential of information (Moss and Townsend 2006). The most remarkable applications of digital media to coordination informatics for pandemic response are likely the creation and activation of global health networks, mandated to surveil infectious diseases internationally and alert regarding their outbreak. These networks have at least partially filled a coordination gap for identifying and initiating responses to global pandemics (see Burkle et al. 2012), but coordination in the implementation of specific response remains largely lacking.

Volunteer and Technical Communities (V&TCs) represent another prominent example of the coordination challenges posed by novel actors in humanitarian response. Volunteer communities are composed of thousands of individuals around the globe who are moved to contribute time and energy to humanitarian response efforts virtually and remotely, often by collecting or processing humanitarian information such as incident reports or spatial data over social media. Though different communities vary significantly in their membership, degree of organization and formal relationships with humanitarian coordinating bodies, their integration into a field marked by seasoned professional field staff and conservative information management is consistently marked by cultural and institutional tensions (Harvard Humanitarian Initiative 2011). Officially and institutionally this is visible in the challenges that surround the development of ethical codes of conducts for digital humanitarians (Meier 2015: 45-60; Resor 2016) or the development of an activation protocol through which OCHA is to include the Standby Task Force (SVT, one of the most prominent V&TCs) in humanitarian response operations (Burns 2014; Gorp 2014).

In Ebola response, V&TCs were particularly prominent in establishing novel information exchange platforms, such as a skype channel for coordinating data collection and identifying data gaps (Fast and Waugaman 2016: 84–88), though there is some evidence that lack of integration into formal coordination mechanisms actually “contributed to gaps in awareness of existing tools and duplication of effort” (ibid 86). This dynamic recalls of other instances where the introduction of digital tools complicates rather than facilitates humanitarian efforts (Bui et al. 2000; Miller et al. 2005; Saab et al. 2008), and is consonant with the more general assessment that the uncoordinated introduction of novel actors into Ebola response exemplifies “wider dysfunction in the provision of global health security” (Harman and Wenham 2018: 10).

Tapia et al.’s (2012) two case studies on humanitarian coordination offer a possible explanation for the failure of information systems to lead to enhanced coordination despite explicit efforts. On the basis of a careful literature review and analysis of two humanitarian coordinating bodies, they examine “the instrumental use of IT as a mechanism by which NGOs collaborate” (ibid 253) and identify an important distinction between IT-driven coordination efforts that are conceptualized as technical or informational challenges, and those that are conceptualized as organizational or process challenges, which they describe as more formidable. Tapia et al. conclude that coordination efforts with modest goals and modest demands on organizational processes are likely to be more successful and increase opportunities for successively more progressive coordination efforts (ibid 253). According to this logic, it would be reasonable to expect that digital media coordination would be most successful when it did not demand changes in organizational practice (i.e., coordinating actors who are already inducted in the use of specific media and coordination efforts that do not require changes to data formats or data collection procedures).

## Novel tendencies in new informatics

This section describes two novel tendencies revealed by the above distinctions: the influx of novel actors that accompany and drive the use of new technologies in pandemic response, and the tendency of new technologies to compound previously distinct activities and workflows within single platforms or single institutional operations.

### Influx of novel actors

New informatics introduce a host of actors and intermediaries not traditionally included in pandemic response and coordination. The ways in which this influx interacts with traditional structures for pandemic response can be considered according to a sandwich model, in which interaction is introduced from above and from below. The bottom slice of the sandwich in this metaphor is interaction with affected populations, where novel informatics present at least two types of challenges.

Firstly, social media and big data introduce promising new sources of information on which to base decision-making in pandemic response, but for whose meaningful use humanitarian organizations tend to lack the institutional and technical capacity, and national authorities even more so (Harvard Humanitarian Initiative 2011; Odugleh-Kolev 2014; Smith 2015; Read et al. 2016). Simultaneously, the participatory ethos of new technology encourages humanitarian organizations and government authorities alike to deliberately engage affected communities in the design, implementation and evaluation of humanitarian response (Kaiser 2000; Maxwell et al. 2011; Gillman 2014; Özdamar and Ertem 2015), yet poses a number of non-trivial hurdles to meaningful engagement. Here too, technical capacities tend to be weakest with national governments, necessitating partnership with international organizations to invest in participatory interventions. Infrastructural requirements and issues of access and representativity can also frustrate intentions to utilize participatory technologies. In a humanitarian context, political realities can often be the most meaningful obstacle, even when all issues of capacity, infrastructure, and access are surmountable. For all these reasons, national authorities are rarely in a position to unilaterally dictate the ways in which new technologies are leveraged to interact with affected populations.

Informatic challenges at the top end of the sandwich arise from the (sometimes unsolicited) engagement from novel international actors. Of particular note are V&TCs and digitally native civil society organizations that are small, nimble, and eager to disrupt established practice. These actors present fundamental challenges to humanitarian coordination by their very engagement. Because they do not fit neatly into traditional humanitarian coordination mechanisms, yet tend to demand attention and heighten expectations, novel international actors at the top level of pandemic informatics are a powerful force for asserting knowledge politics in informatics of response. The information flows described by Fast and Waugaman illustrate not only information exchanges in this sense, but fundamental assertions and negotiations about what kind of information is relevant and where and by whom those decisions are made. The moments at which this happens are not regulated by traditional policies of humanitarian coordination or cluster mechanisms, but occur in the practical application of technologies to knowledge and information, what some scholars have termed moments of closure in humanitarian knowledge politics (Burns 2014).

In some instances, international informatics are efficiently structured to serve national authorities. The introduction of data and information clearing houses presents opportunities for national authorities to assert control of national agendas, for example, such as when the Humanitarian Data Exchange enabled Guinean ministries to track training of infection prevention and control training efforts in the country (Fast and Waugaman 2016: 90). This dynamic appears exceptional in a chaotic response environment marked by a “myriad of actors with no clear role or leadership” (Harman and Wenham 2018: 10), however. A pandemic response context where “many of the information collection systems that organizations set up during the response were not linked to national systems or national capacity” (56) necessarily reinforces the capacity and agency of international actors, and often novel actors, at the expense of national authorities’ influence over response processes.

It is also worth noting informatics practices that transcend this two-level model. Most clearly in opposition to the agency of national authorities is the introduction of “hidden actors”, such as application developers, producers of hardware or network managers with de facto influence over humanitarian data during its collection and processing (see Gillman 2014: 7). Equally notable are instances in which international actors bypass engagement with the traditional response environment altogether. In some instances, this appears to occur exclusively at the international register, with little or no contact with any in-country actors. Examples include international networks of scientists collaborating to improve diagnostic tools (Eclipse 2017), or hackathons organized in foreign capitals to develop data models or applications for implementation in pandemic response (Sangokoya 2014; Gordon 2016; Lodato and Di Salvo 2016). Such initiatives raise serious questions about opportunity cost and efficient use of resources.

To summarize, digital communication technologies and information flows introduce a host of novel actors to pandemic response. Traditional humanitarian actors may experience this at the top level through the novel engagement of international actors, or at the bottom level through pressure to engage with affected populations and their data. In each instance, the influx of novel actors carries a risk for decision-making authority and agency to be moved into novel fora, further outside the influence of national authorities.

This may not occur in an absolute sense; indeed, the role of national governments in driving Ebola response strategies has been significant (DuBois et al. 2015: 21). Yet the potential for exclusion merits careful consideration, particularly given the already pronounced tendency for agency of national authorities to be limited by the militarization of humanitarian response (Sandvik 2015) and the increasing prominence of international NGOs in public health service provision (Prince 2014). Such dynamics are particularly vulnerable in the contexts of pandemic and crisis response, and reviews of the Ebola response have also noted how decision-makers’ identities shape response strategies (DuBois et al. 2015: 27).

The application of digital technologies has undoubtedly opened up a host of opportunities for decision-making by novel actors. This may occur in situations where national authorities enjoy a limited role, such as the network communications of global watchdog networks in which NGOs, scientific communities, international health networks and government agencies contribute to multiple digital communications streams and daily webinars to coordinate disease surveillance and response (Ramalingam 2015: 11–13). It may also occur in digital for where there is no direct participation by national authorities, or in situations where national authorities lack the basic technical capacities to engage in a natural coordinating role, such as when “a host of academics, private philanthropists and technology companies” lobbied telecom companies for access to call detail records in order to develop their own response strategies (Sandvik et al. 2017: 16).

Though there are some cases in which novel actors and information practices support a stronger role for national authorities in pandemic response and a greater capacity to exercise agency and decision-making in the design and implementation of that response (Ramalingam 2015: 10-14; Fast and Waugaman 2016: 63-66), but this appears rare and the conditions under which it occurs are unclear. The overwhelming picture is one in which technologically driven informatics exacerbate coordination challenges (Kim 2014), driving the enactment of knowledge politics outside formal structures of humanitarian clusters and beyond the influence of national authorities.

### Convergence

Though the boundaries between diagnostic activities, risk communication, and response coordination were perhaps never entirely clear, these areas have traditionally been institutionally and procedurally distinct. Digital communications’ affordances and functionalities make it increasingly possible to combine activities from these areas in single processes.

This recalls theories of technological convergence, in which the increasing capacity of media tools to integrate multiple functionalities corresponds with broader shifts in markets and genres (Kim 2014). As described by Henry Jenkins (Huerta and Tsimring 2002):

Our cell phones are not simply telecommunications devices; they also allow us to play games, download information from the internet and receive and send photographs or text messages. Any of these functions can also be performed through other media appliances. One can listen to The Dixie Chicks through a DVD player, car radio, walkman, computer MP3 files, a web radio station or a music cable channel. Fueling this technological convergence is a shift in patterns of media ownership. Whereas old Hollywood focused on cinema, the new media conglomerates have controlling interests across the entire entertainment industry (34).

A detailed exploration of how these dynamic maps onto humanitarian technology exceeds the scope of this article, but we feel justified in arguing that there are at least two comparable dynamics. First, we will use the term *digital* convergence to refer to the ways in which technology enables a concentration of diverse tasks in single platforms and workflows (the same platform conducting a range of tasks). Second, the examples cited in this article consistently exemplify how this type of convergence is coincident with what we term *operational* convergence, whereby specific types of information and communication management tasks are distributed across novel institutional and organizational groups of actors (same tasks conducted by a range of actors). Below, we briefly describe four examples that demonstrate how this can occur in pandemic response. These examples are not explored in depth, but are meant to illustrate the consistent interplay of convergence with digital media and the injection of novel actors across a variety of humanitarian settings.

The first example is what Ramalingam terms “watchdog and knowledge networks” (2015), understood as networks involved in the “early detection of disease, characterization of the disease, and subsequent reporting and communication directed to decision makers in governments, international bodies and other key audiences” (ibid 9–10). Especially instructive is Ramalingam’s analysis of GOARN, the Global Outbreak Alert and Response Network. As an “operational arm” of the WHO and network of networks that together surveil the outbreak and spread of infectious diseases, GOARN functions as a de facto coordination mechanism for international and national actors engaged in pandemic response. The scope of GOARN’s activity in this regard is impressive, having responded to over 100 outbreaks in over 50 countries between 2000 and 2015, and the network has played a crucial role in the SARS outbreak in 2003 and the avian influenza outbreaks of 2004. Notably, the efficiency and scope of GOARN’s activities is explicitly attributed to the fact that coordination and information exchange occur

across a diverse digital infrastructure that supports text messaging, email, and web-based applications, all of which are employed in tandem to ensure the right knowledge and information get to where they are needed at the right time, and importantly, allows a two-way exchange of information across the network (11).

Notably, the diverse communications between national authorities, WHO staff, NGOs, and scientific institutions are consolidated in a coherent institutional framework and through weekly webinars. This process interoperates diagnostic and coordination, feeding them directly into the establishment of response protocols, including protocols for risk communication, which are in many instances executed by the same actors that provided diagnostic information, by virtue of their capacity for rapid, two-way communication with populations.

Though it is unclear the degree to which this operational convergence of such tasks diminishes the agency of national authorities in a general sense, it is reasonable to expect that the inclusion of multiple actors decreases governments’ scope for top down control. This may often be for the better in terms of effective response, as is likely the case with SARS in 2013, when GOARN’s access to digital communications platforms facilitated the supply on non-governmental diagnostic information, which likely contributed to acknowledgement of the outbreak by the Chinese government.

*Assisted Contact Tracing* provides a second example. Contact tracing is the epidemiological practice of identifying the individuals who have come into contact with infected individuals, in order to map the spread of a disease (Huerta and Tsimring 2002). In 2014, a private company named Odisi developed a platform for “Assisted Contact Tracing” (ACT), which digitized this process through the use of Integrated Voice Recognition Software. Individuals in Ebola-affected communities were able to report their contacts using mobile phones and then received follow-up messages regarding care and updates on the Ebola response. This digitized approach increased the efficiency of data collection by eliminating the need for human interviewers, and also allowed the integration of other types of data (paper and mobile data), which increased the platform’s diagnostic capacity dramatically. Though the platform was designed and implemented as a diagnostic tool, the affordances offered by digital media quickly presented other opportunities. The automated registry of exposed individuals was quickly adapted for risk communication purposes via SMS follow-up messages, and the digitization of rapid analysis of data promptly positioned the ACT platform to play a coordinating role among parallel diagnostic initiatives. Here, we see a clear digital convergence of diagnostic and risk communication activities on a mobile phone platform, at the discretion of a private company.

A third example is offered by U-Report, a free *SMS-based polling tool* launched in Uganda in 2011 through a civil society partnership in order to monitor the quality of human rights and governance in the country, with a focus on polls related to human rights. In early 2012, platform users noted early signs of the outbreak that would later come to be known as “nodding sickness” and would claim over 3000 lives in the following months. U-report did not have a health mandate and did not solicit these early epidemic reports, but received them because the communication platform was already in place and integrated into the communication habits of users. In this sense, the presence of a digital media infrastructure very much conditioned the implementation of a diagnostic tool, which promptly provided a site for national coordination, as U-Report collaborated with the Ministry of Health and the WHO to develop and implement a 4-stage communications and mobilization plan. Here again we see the digital and operational convergence of diagnostic activities and risk communication activities by national authorities with the support of international organizations. Notably, while national authorities are directly engaged in both sending and receiving content, they do not have direct control of the operational and financial processes that support the initiative.

Fourth and lastly, the *Humanitarian Data Exchange* (HDX) was established by the UN Office of Coordination for Humanitarian Affairs in 2014, in an effort to improve and coordinate access to humanitarian data. The Ebola Crises Page collected 62 data sets from UN, governmental, civil society, and private sector data sources and invited users to contribute their own data to the site. The page also featured maps and visualizations developed in collaboration with private charitable foundations and private businesses. As such, the site represents a near seamless integration of all the components of pandemic informatics. It directly served the needs of independent diagnostic efforts by providing access to quality humanitarian data; it performed a coordinating function by establishing standards and expectations for the use and production of humanitarian data, and visualizations and graphics created by the community were incorporated into the HDX gallery for download and use in independent risk communication efforts. Here, we see convergence between digitally enabled coordination efforts, deeply rooted in multilateral humanitarian institutions, and the diagnostic processes that they enable. Unlike the examples above, the question of who engages with the HDX and how is not significantly pre-determined. The platform is designed to be open, and to the extent that contributors are invited and approved by OCHA, it is reasonable to expect that it is open to national authorities. To the extent that technological capacity still limits national authorities from engaging with the platform, convergence of diagnostic and coordination activities nonetheless consolidates what we can call informatic discretion outside their spheres of influence.

What is striking about these examples is not necessarily the fact that activities related to different informatic components interact; that has to some degree perhaps always been the case. What is striking is the degree to which they do so automatically, as conditioned by digital media, and within the purview of the actor driving the technological and informatic innovation. This is almost never national authorities, due to capacity issues described above. To the extent that reliance on technology and the introduction of novel actors drive informatic discretion beyond the influence of national authorities, this phenomenon is likely to be exacerbated by instances of technological and operational convergence.

## Conclusions and recommendations

This analysis has reviewed the ways in which digital technologies have been deployed in humanitarian pandemic response, and proposed a three-part conceptual model for assessing these informatics according to the objectives that are pursued. Doing so revealed two consistent and interrelated consequences:

1. The *influx of novel actors* from both affected populations and from the international register engenders novel fora for asserting knowledge politics, influence, and informatic discretion in response. These fora support the increased engagement of novel actors, but are often inaccessible to national authorities due to limits on technical capacity and political position.
2. The efficiency of technologically driven informatics tends towards *technological and operational convergence*, in which multiple types of activities are collapsed into single platforms or institutional processes. Convergence has the effect of further consolidating sites for informatic discretion beyond the influence of national authorities.

These two dynamics are concomitant and mutually reinforcing. Each is directly enabled by the introduction of novel technological tools and strategies to pandemic response, but also facilitates the other (novel actors tend to be the ones who actually combine informatics functionalities, often without warning and through their own innovations), and complicates traditional pandemic response informatics in mutually reinforcing ways. It is tempting to anticipate a certain momentum at play.

It is also tempting to frame this as a coordination problem. Novel actors and models for participation strain the humanitarian system precisely because humanitarian roles and responsibilities are rigidly defined, but not immutable. The dynamics of convergence would, moreover, seem to promise significant increases for efficiency and coordination were obstacles to information sharing surmounted, and gaps in institutional cultures for using technology filled. But herein lies the challenge. Though technology seems consistently to imply dramatic gains in humanitarian efficiency, it consistently frustrates efficient coordination, and despite millions of dollars spent, in the case of multi-lateral, large-scale coordination efforts around information, there have only been several very public failures (Tapia et al. 2012: 240).

This is likely because the challenge manifest in these informatics is more fundamental, having to do with the ways in which institutions and organizations leverage technologies to assert knowledge politics “in ways that rely upon the differential influence and authority that is granted to particular forms of knowledge or representations” (Barnett 2011). Without addressing the differential capacities and expertise that drive a propagation of knowledge politics beyond national authorities’ spheres of influence, coordination exercises are unlikely to be anything more than exercises. In this scenario, a humanitarian technological context marked by exuberant expectations and a chaotic lack of coordination is problematic. More troubling is the underlying recession of national authorities from those fora in which knowledge and influence are asserted and contested.

The tendency of international humanitarian response to sideline national authorities is not new. The possible exacerbation of this dynamic by new response informatics is, however, particularly problematic because the enthusiasm surrounding novel technologies so efficiently occludes the challenges that they pose to fundamental coordination norms of humanitarian coordination. There is wide agreement that international humanitarian intervention should complement and support national authorities’ response as a temporary measure until the point at which national authorities are able to assume control over national processes, facilities, and infrastructure (Jahre and Jensen 2010; Harvey and Harmer 2011; OCHA Inter-Agency Standing Committee 2015). The details of this relationship prompt arduous contention and debate in the context of traditional humanitarian coordination mechanisms, such as the UN cluster system (Harvard Humanitarian Initiative 2011; Sandvik et al. 2014; McDonald 2016), and to a modest degree, in critical commentary on the application of humanitarian technology (Sandvik et al. 2017). Until they are equally visible in the discourses and planning processes that drive remote volunteering, university hackathons, mobile network-enabled contact tracing, and participatory mapping efforts, it is hard to imagine ways in which to reassert the agency and influence of national authorities in new response informatics.

So what is next? There are at least three opportunities to begin addressing this. Firstly, the degree to which digitally driven informatics exacerbate challenges to the agency of national authorities in the context of humanitarian coordination should be explicitly included in critical discourses that resist techno-optimism in the humanitarian sector. This involves expanding notions of responsible humanitarian technology, innovation and data-use to include reflections on how digital informatics impact the spheres of influence of national authorities in humanitarian response. It constitutes an additional type of risk to be considered when questioning the risks associated with humanitarian experimentation (Harvey and Harmer 2011).

Secondly, established mechanisms for coordinating complementary and supplementary humanitarian support to national authorities in humanitarian response, including the UN cluster system, should deliberately anticipate these dynamics and work to mitigate their effects. Significant work is ongoing to improve the ways in which national and international actors interact in humanitarian response (UNGA 1991; OECD 2008; Jahre and Jensen 2010; Odugleh-Kolev 2014). The policies and procedures that result from these efforts should include explicit processes for accommodating the types of novel actors, platforms and instances of convergence described above. Explicitly identifying roles and sites for interaction with national authorities would have the twin benefit of providing entry points for those actors according to established coordination norms, and providing opportunities for technical capacity development to national authorities.

Lastly, prominent humanitarian coordination bodies that have significant experience using digital communication technologies and interacting with the novel actors they introduce, should facilitate structured interaction between these actors and representatives of national authorities, in an effort to identify good practices for reinforcing national agency in digitally-driven informatics. Taken to their logical conclusion, relatively low-cost investments in events and joint trainings by organizations such as OCHA, could easily lead to the production of credible and welcome guidelines for novel actors at the top of the informatics sandwich, which include an explicit role for national authorities, protecting their agency and influence in the design and implementation of response.

This agency matters. A clear and meaningful role for national authorities is worth safeguarding in order to ensure efficient and appropriate response (UNGA 1991; OECD 2008; Jahre and Jensen 2010; Odugleh-Kolev 2014), but also because it is essential for securing the sustainability and long term impact of health outcomes in the wake of pandemic response (Odugleh-Kolev 2014;(DuBois et al. 2015). The conceptual model and critical analysis proposed here provide a preliminary lens with which to purse those ends.

## Abbreviations

ACT: Assisted Contact Tracing
GIS: Global information system
GOARN: Global Outbreak Alert and Response Network)
HDX: Humanitarian Data Exchange
NGOs: Non-governmental organizations
OCHA: Office for the Coordination of Humanitarian Affairs
ProMED: Program for Monitoring Emerging Diseases
SMS: Short messaging system
SVT: Standby Volunteer Task Force
UN: United Nations
UNICEF: United Nations Children’s Fund
WHO: World Health Organization
